# An Evidence Map on Serious Games in Preventing Sexually Transmitted Infections Among Adolescents: Systematic Review About Outcome Categories Investigated in Primary Studies

**DOI:** 10.2196/30526

**Published:** 2022-02-02

**Authors:** Karina Ilskens, Kamil J Wrona, Christoph Dockweiler, Florian Fischer

**Affiliations:** 1 School of Public Health Bielefeld University Bielefeld Germany; 2 Faculty of Health University of Applied Sciences Bielefeld Bielefeld Germany; 3 Department of Digital Public Health and Biomedicine School of Life Sciences University of Siegen Siegen Germany; 4 Institute of Public Health Charité - Universitätsmedizin Berlin Berlin Germany; 5 Institute of Gerontological Health Services and Nursing Research Ravensburg-Weingarten University of Applied Sciences Weingarten Germany; 6 Bavarian Research Center for Digital Health and Social Care Kempten University of Applied Sciences Kempten Germany

**Keywords:** serious games, entertainment education, STI, STD, sexual health, effect, impact, sexually transmitted infections, adolescents, adolescent sexual health

## Abstract

**Background:**

Sexually transmitted infections (STIs) represent a global health risk. Adolescents are at increased risk of infection for several reasons such as lack of knowledge, risky sexual behaviors, and lack of behavioral sills (eg, to negotiate safer sex). Given the fact that adolescents often use digital media and that serious games are considered to have the potential to change knowledge, attitudes and behavior, serious games represent an opportunity for the prevention of STIs.

**Objective:**

The aim of this systematic review was to identify and systematically summarize the dimensions that have been investigated in primary studies on serious games targeting STI prevention among adolescents.

**Methods:**

A systematic review was conducted in PubMed and Web of Science. Studies published from 2009 to 2021 were included that assessed the effectiveness of serious games on adolescent sexual health. A total of 18 studies met the inclusion criteria and were categorized according to dimensions of effectiveness and user experience.

**Results:**

Various dimensions of effectiveness and aspects of user experience were investigated in the primary studies. In total, 9 dimensions of effectiveness were observed: sexual behavior, behavioral intentions, knowledge, attitudes and beliefs, self-efficacy and personal limitations, character traits and future orientation, environmental and individual risk factors, risk perception and risk assessment, as well as normative beliefs and (social) norms. Furthermore, several dimensions related to user experience were investigated in primary studies, that is, motivation, acceptability, trustworthiness, comprehensibility, handling and control, perceived effectiveness, as well as satisfaction.

**Conclusions:**

This review provides an overview of serious games interventions that are vastly different in approach, content, and even platform. In previous studies, knowledge has already been comprehensively assessed, and a positive influence of serious games on knowledge about sexual topics is evident. The results clearly show that adolescents’ sexual knowledge has been increased by the serious games interventions. However, methodological and content differences in the surveys make it difficult to draw conclusions about the effectiveness related to changes in attitudes and behavior.

## Introduction

From a global perspective, the relevance of sexually transmitted infections (STIs) is reflected in continued high numbers of HIV infections, particularly in African countries [1,2] and the fact that approximately 1 million people worldwide become infected with a curable STI every day [3,4]. In addition to specific risk groups such as men who have sex with men, sex workers, and intravenous drug users [5-9], adolescents are an important target group in STI prevention approaches. For example, increasing rates of infections among adolescents have been found for chlamydia in recent years [10,11]. Although studies indicate that knowledge of HIV has increased, knowledge of other STIs is significantly lower, even despite their widespread [12,13]. Misinformation as well as knowledge gaps related to STIs frequently occur among adolescents from poor to middle class backgrounds [14]. Lack of knowledge and low awareness of STIs as well as shame and fear inhibit adolescents from talking about STIs and contacting health care providers when problems arise [15]. These conditions may increase risky sexual behaviors and, thereby, the risk of acquiring an STI [16].

As a result of the high prevalence of STIs among adolescents and frequent use of digital media in this group, there is increasing consideration of using digital approaches as part of sexuality education and STI prevention in educational settings [17,18]. One of the instruments being discussed are digital games. Popularity and interest in these serious games have been growing in research and practice in recent years [19]. Serious games are an instrument of entertainment education, which include “any attempt to make learning [more] enjoyable, no matter if media-based, mediated or within a classroom setting” [20]. Various definitions for serious games exist, but all definition have, in common, to focus on the serious use of games to achieve goals, such as learning and education, by combining serious topics with entertaining and, in recent times frequently, multimedia aspects [21]. These serious games are challenging and engaging and supply the users with competencies useful in reality [22]. Therefore, serious games are characterized particularly by the use of a competency-based approach by integrating the game principle into the learning process and thus becoming part of this process [23]. Overall, digital game applications are applied with the aim of increasing attention and knowledge as well as changing attitudes and behavior [21]. Areas of disease prevention and health promotion for the application of serious games are comprehensive sexuality education (CSE), STI prevention, and promotion of sexual health [19,24]. The potential of serious games to positively influence knowledge, attitudes, and behaviors and to promote attention are goals that are also pursued in CSE [17]. Serious games, with their innovative character, thus form a new way to educate adolescents about sexual health and, thereby, contribute to STI prevention.

The characteristics of serious games offer manifold possibilities for STI prevention, which make their use reasonable. First, the entertaining nature of serious games offers the opportunity to facilitate communication about sensitive topics such as sexuality and sexual health [19,25]. Second, serious games allow to keep content anonymous and confidential, making it easier to deal with these sensitive topics [24]. Third, through the digital, fictional, and playful environment, serious games offer a low-threshold access and usage as well as the possibility to have different experiences without being exposed to real risk [26]. Fourth, the content of serious games can be adapted to the demands and requirements of the target group as a whole and even at an individual level through tailored approaches [24]. Owing to gender-, origin-, and culturally-specific differences as well as various sexual experiences and orientations, serious games offer great potential in STI prevention [24,26,27]. However, criticism related to serious games is expressed in that the content, target groups, as well as the quality of the games greatly differ, and existing evaluation studies are inconclusive. In studies on the effectiveness of serious games, the focus is on the measurement of so-called “soft facts,” which are collected, for example, through self-reported competence beliefs. Therefore, the results need to be evaluated with caution [21].

In order to implement serious games in practical work with adolescents, it is important to investigate whether and how serious games influence adolescents and impact STI prevention. This evidence map [28] based on a systematic review focusses on the outcomes, which have been investigated in previous primary studies related to serious games targeting STI prevention. The aim is not to summarize the direct effects of respective serious games, which are also quite heterogeneous in terms of approach and content, but to provide a systematic overview about the outcomes (effectiveness/impact and perceptions/user experience) that have been investigated to date.

## Methods

We conducted a systematic literature review in 2 databases, namely, MEDLINE (via PubMed) and Web of Science to identify studies investigating serious games in the context of STI prevention, which have been published until March 2021. The search in PubMed was conducted in May 2019 and an update was performed in March 2021. The search in Web of Science was conducted in March 2021. The search strategy consisted of a combination of terms related to the type of the game with the field of action. For both databases, we used the following complete search algorithm:

(game* OR video game* OR interactive multimedia OR serious game*) AND (sexual health OR sexual transmitted infections OR sexual transmitted disease OR sexuality education OR hiv OR sti OR sexuality*)

In PubMed, we applied the filters to include only those studies published since 2009 because the aim was to identify only recent literature (published in the past decade) owing to the fast progress in the development of serious games. Furthermore, a filter was applied to restrict the search to studies published in English or German language. In addition, we used a backward snowballing technique by searching the reference list of studies included in the full-text screening.

To identify appropriate studies, 2 reviewers screened the studies with regard to their (1) title and abstract and (2) appraised the full-texts if inclusion criteria were fulfilled. The basic criteria for the combined screening of title and abstract was whether the information provided seemed to be related to serious games related to preventing STIs. Overall, 1559 manuscripts were identified after removal of duplicates. To ensure systematic management of the information, references located through the search were downloaded to a bibliographical software package (Citavi 6, Swiss Academic Software), which automatically identifies and removes duplicates.

**Figure 1:**
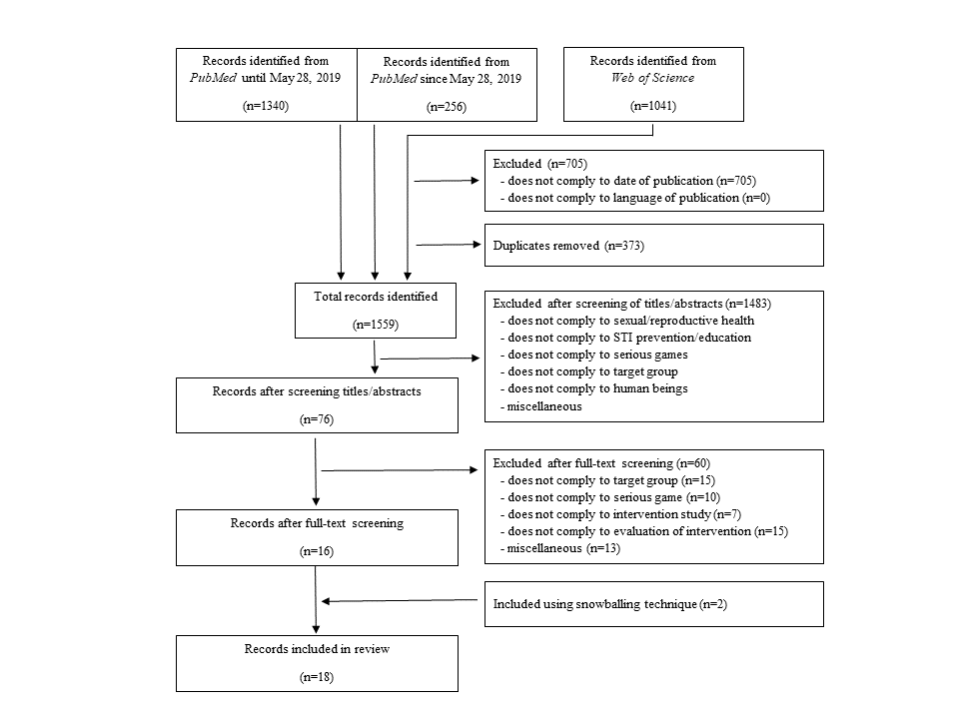
Flowchart for study selection. STI: sexually transmitted infection.

Two authors (KI and KJW) carried out the title and abstract screening. Subsequently, KI and KJW independently reviewed the full texts (n=76) to determine whether the inclusion criteria were met. We defined inclusion and exclusion criteria (Table 1), which were used for the screening of full texts. According to this, studies that did not comply with serious games (n=10) or the target group of adolescents (n=15) were excluded. Furthermore, we excluded studies that did not describe an intervention study (n=7), did not comply with the evaluation of an intervention (n=15), or for further reasons (n=13) such as a focus on individuals who tested positive for an STI. There were no divergent appraisals between the 2 reviewers. From a total of 76 relevant studies (and 2 additional studies identified through the snowballing technique), 18 were identified for inclusion in this overview (Figure 1). Overall, the search algorithm used in PubMed proved to be very reliable, because the supplementary search in Web of Science led to only 1 additional record included in the synthesis.

We analyzed whether serious games have been used for STI prevention and CSE and mapped the results according to the levels of effectiveness/impact and perceptions/user experiences of users. The results are described in form of a qualitative overview, allowing for a systematization of the outcomes, which have been addressed in previous studies so far. We did not perform a quality appraisal of primary studies because various study designs (eg, randomized controlled trial [RCT], quasi-experimental study, pilot test) have been included and no meta-analysis was performed. The procedures and reporting of the systematic review follow the recommendations published in the PRISMA (Preferred Reporting Items for Systematic Reviews and Meta-Analyses) statement [29].

**Table 1:** Inclusion and exclusion criteria for screening of full texts.

Criterion	Inclusion	Exclusion
Population	Adolescents (9-21 years)	Adults; particular risk groups (eg, men who have sex with men, drug users, sex workers); sexually transmitted infection/HIV-positive persons
Study design	Empirical study including an evaluation of a serious game	Studies of theoretical nature only with no empirical data
Type of study	All published studies, identifiable via PubMed or Web of Science Abstract must be available	Books; all forms of grey literature, including conference abstracts, commentaries, presentations, proceedings, regulatory data, unpublished trial data, government publications, dissertations/theses, journalistic interviews, policy reports as well as any other nonscientific material No abstract available
Focus of study	Sexually transmitted infections Comprehensive sex education or HIV/ sexually transmitted infection prevention	Nonsexually transmitted Noncomprehensive sexuality education or HIV/sexually transmitted infection prevention Other aspects in a broader sense of sexual health (eg, sexual violence, sexual dysfunction, use of antiviral therapy or HIV pre-exposure prophylaxis) not related to sexually transmitted infection prevention in the general population using digital technologies
Role of digital serious game	Digital serious games (including all digital games for learning purposes)	No use of digital serious games Analogous serious games or gamification approaches
Language of publication	English and German	All other languages other than English or German
Date of publication	January 2009 to March 2021	Before 2009

## Results

### Characteristics of the Studies

A total of 18 studies identified within the systematic review were included in the synthesis on effectiveness and user experiences investigated in primary studies related to serious games in STI prevention (Table 2). Three of the studies are usability tests that focus on usability, user experience, and cultural adaptation or adaptation, among other factors [30-32]. One of these studies only reported perceived efficacy [31]. Another study included a usability and a feasibility study, in which adapted computer-based components of an existing intervention are tested, and subsequently, a computer-only intervention developed based on these findings is deployed [33]. The aforementioned studies do not focus on efficacy, but they do provide evidence of effectiveness and insights into the play experience. For that reason, they were included in this systematic review. In total, 10 completely different serious games interventions have been focused upon in primary studies. The intervention that was used most frequently in the studies was “It’s Your Game”—it was used in 10 out of 18 studies. In each of the other studies, different interventions were used. Overall, 8 studies have been conducted as RCTs. Interventional studies used either a conventional health training or video games as controls. The target group consisted entirely of schoolchildren and teenagers between the ages of 12 and 19 years. Various study designs and methods have been used. The sample size ranges from 19 to 4562 participants. Table 2 presents the main information about the primary studies. Further details about the characteristics of the interventions are described in Multimedia Appendix 1.

Various dimensions of effectiveness and aspects of user experience were investigated in the primary studies. At least one dimension of effectiveness has been addressed in 16 of the 18 studies. In total, the following 9 dimensions of effectiveness were observed: (1) sexual behavior, (2) behavioral intentions, (3) knowledge, (4) attitudes and beliefs, (5) self-efficacy and personal limitations, (6) character traits and future orientation, (7) environmental and individual risk factors, (8) risk perception and risk assessment, and (9) normative beliefs and (social) norms.

Furthermore, 7 aspects related to user/gaming experience were investigated in overall 11 studies: (1) motivation, (2) acceptability, (3) trustworthiness, (4) comprehensibility, (5) handling and control, (6) perceived effectiveness, and (7) satisfaction. A full overview of the categorizations can be found in Table 3 and Table 4. 

**Table 2:** Characteristics of the primary studies.

Authors, year of publication, country	Intervention	Study participants (n)	Age (years; range)	Study design	Control group	Methods	Follow-Up
Bertozzi et al [34], 2018, India	My future family	393	15-19	Pilot study		Questionnaire (paper-and-pencil based, postgame) In-game data	
Chib [35], 2011, Peru	Planeta Riesgo X	102	15-18	Quasi-experimental panel design		Questionnaire (self-administered)	
Chu et al [36], 2015, Hong Kong	Making Smart Choices	1176	12-16	Pilot experiment with pretest and posttest		Knowledge: Questionnaire Feedback: Questionnaire and focus group interviews	
Escobar-Chaves et al [30], 2011, Puerto Rico	It’s Your Game: Keep It Real	73	12-14	Pilot study		Questionnaire (paper-and-pencil based) Class discussion Reactions and opinions from 2 teachers, a librarian and a social worker	
Fiellin et al [37], 2017, USA	Play Forward: Elm City Stories	333	11-14	Randomized controlled trial	✓	Questionnaire (face-to-face assessments) In-game data	✓
Gariepy et al [38], 2018, USA	Mobile videogame intervention	26	15-17	Pilot study		Questionnaire (self-administered) Focus group discussions	✓
Haruna et al [39], 2018, Tanzania	Game-Based Learning	120	11-15	Randomized controlled trial	✓	Questionnaire based Focus group interview	
Markham et al [40], 2012, USA	It’s Your Game: Keep It Real	1258	mean 12.6	Randomized controlled trial	✓	Audio-computer-assisted self-interview	✓
Oliveira et al [41], 2016, Brazil	Papo Reto	23	15-18	Qualitative study (descriptive and exploratory)		Recorded workshops and speeches analyzed by content analysis	
Peskin et al [42], 2015, USA	It’s Your Game: Keep It Real	1374	mean 14.3	Randomized controlled trial	✓	Audio-computer-assisted self-interview	✓
Peskin et al [43], 2019, USA	It’s Your Game: Keep It Real	1543	mean 13	Randomized controlled trial	✓	Audio-enhanced, computer-assisted surveys	✓
Potter et al [44], 2016, USA	It’s Your Game-Tech	3143	mean 12.7	Randomized controlled trial	✓	Audio-enhanced, computer-assisted surveys	✓
Rohrbach et al [45], 2019, USA	It’s Your Game: Keep It Real	4562	mean 15	Quasi-experimental cohort study		Audio-enhanced, computer-assisted surveys	✓
Shegog et al [33], 2014, USA	It’s Your Game-Tech	33, 22	12-14 13-15	Single-group, pre/postusability test		Computer-based questionnaire	
Shegog et al [31], 2017, USA	Native It’s Your Game	45	11-15	Usability test (preadaption, adaption, postadaption)		Questionnaire (paper-and-pencil based) Focus group discussions Community advisory meetings	
Shegog et al [32], 2021, USA	Secret of Seven Stones	19	mean 12	Usability test		Questionnaire Qualitative interviews	✓
Tortolero et al [46], 2010, USA	It’s Your Game: Keep It Real	981	mean 13	Randomized controlled trial	✓	Audio-computer-assisted self-interview	✓
Winskell et al [47], 2018, Kenya	Tumaini	60	11-14	Randomized controlled trial	✓	Audio-computer-assisted self-interview For intervention arm: Postintervention survey Postintervention focus group discussions	✓

**Table 3:** Dimensions of effectiveness.

Study	Sexual behavior	Behavioral intentions	Knowledge	Attitudes and beliefs	Self-efficacy and personal limitations	Character traits and future orientation	Environmental and individual risk factors	Risk perception and risk assessment	Normative beliefs and (social) norms
Bertozzi et al (2018) [34]			✓	✓					
Chib (2011) [35]			✓	✓	✓				
Chu et al (2015) [36]			✓	✓					
Escobar-Chaves et al (2011) [30]			✓						✓
Fiellin et al (2017) [37]	✓	✓	✓	✓					
Gariepy et al (2018) [38]		✓	✓		✓		✓	✓	
Haruna et al (2018) [39]			✓						
Markham et al (2012) [40]	✓	✓	✓	✓	✓	✓	✓		✓
Oliveira et al (2016) [41]			✓				✓		
Peskin et al (2015) [42]	✓	✓	✓	✓	✓	✓	✓		✓
Peskin et al (2019) [43]	✓	✓	✓	✓	✓		✓		✓
Potter et al (2016) [44]	✓	✓	✓	✓	✓		✓		✓
Rohrbach et al (2019) [45]	✓		✓	✓	✓	✓	✓		✓
Shegog et al (2014) [33]		✓	✓	✓	✓				✓
Shegog et al (2017) [31]									
Shegog et al (2021) [32]									
Tortolero et al (2010) [46]	✓	✓	✓	✓	✓		✓		✓
Winskell et al (2018) [47]		✓	✓	✓	✓	✓	✓	✓	✓

**Table 4:** Dimensions of aspects of user/gaming experience.

Study	Motivation	Acceptability	Trustworthiness	Comprehensibility	Handling and control	Perceived effectiveness	Satisfaction
Bertozzi et al (2018) [34]	✓					✓	✓
Chib (2011) [35]	✓		✓		✓	✓	✓
Chu et al (2015) [36]		✓				✓	✓
Escobar-Chaves et al (2011) [30]	✓	✓	✓	✓	✓	✓	✓
Fiellin et al (2017) [37]							
Gariepy et al (2018) [38]	✓	✓				✓	✓
Haruna et al (2018) [39]	✓			✓		✓	✓
Markham et al (2012) [40]							
Oliveira et al (2016) [41]	✓			✓			✓
Peskin et al (2015) [42]							
Peskin et al (2019) [43]							
Potter et al (2016) [44]							
Rohrbach et al (2019) [45]							
Shegog et al (2014) [33]	✓	✓	✓	✓	✓	✓	✓
Shegog et al (2017) [31]	✓	✓	✓	✓	✓	✓	✓
Shegog et al (2021) [32]	✓	✓	✓	✓	✓	✓	✓
Tortolero et al (2010) [46]							
Winskell et al (2018) [47]	✓					✓	✓

### Dimensions of Effectiveness

#### Sexual Behavior

The dimension of participants’ sexual behavior has been reported in 7 of the 18 studies. Here, all 7 studies consider the influence of serious games on the delay of first sexual intercourse. Three studies found an impact on delay of the first sexual intercourse in the intervention group. These results refer to anal, oral, and vaginal intercourse [40,45,46]. Six of the 7 studies refer to sexual behavior in the past 3 months, with the following aspects: sexual intercourse in general and its frequency, condom use or contraception, alcohol and drug use, number of lifetime sexual partners, and frequency of unprotected intercourse [40,42-46]. Three studies have observed influences of serious games on individual aspects [40,45,46]. Overall, these studies were able to observe positive effects of the respective serious games on participants’ sexual behavior, although no study showed a positive result for all aspects. Four studies failed to find any effect of the serious game investigated on behavior, although positive trends were found in 1 study [43].

#### Behavioral Intentions

Nine studies focused on the impact of serious games on adolescents’ behavioral intentions. Of these 9 studies, 6 looked at abstinence intention until marriage or school completion [33,40,42-44,46]. Among these, 3 studies observed a positive effect of serious games, although this was found only for abstinence until marriage in 1 study [33] and only for abstinence for high school graduation in 1 study [46]. One study detected a positive effect for both aspects [40]. Three studies found no effect [42-44]. Seven of the 9 studies assessed the intentions to have sexual intercourse [33,37,40,42-44,46]. In this regard, 3 studies found a positive effect of the respective serious games in that participants in the intervention had fewer intentions to have sexual intercourse in the next year [40,43,46].

#### Knowledge

The impact of serious games on sexual knowledge as a key dimension to promote sexual health was investigated in 16 of the 18 studies. In 13 studies, the effectiveness was tested on the dimension of knowledge by using questions on a variety of sexual and reproductive health topics [33,35-40,42-47]. In summary, 12 of the 16 studies found positive effects of the investigated serious games on knowledge related to sexual and reproductive health as well as related topic areas. In 4 studies, the results were related to overall sexual and reproductive health, with all 4 studies finding a positive effect of serious games on participants’ knowledge [37-39,47]. Nine studies related to knowledge dimension regarding specific sexual and reproductive health topics [33,35,36,40,42-46]. Eight of these studies considered the effects of a serious game on participants’ contraceptive knowledge [33,35,40,42-46], of which 7 studies found a positive impact of the respective serious game [33,40,42-46]. Throughout 4 studies, the majority of participants perceived knowledge acquisition following the use of a serious game, providing evidence for a positive effect of the intervention on sexual knowledge [30,34,39,47].

#### Attitudes and Beliefs

Twelve studies examined attitudes and beliefs on sexual behavior and other sexual topics. Seven of these studies investigated reasons for or against performing sexual intercourse [33,40,42-46]. Five of the 7 studies found a positive effect of serious games [33,40,44-46]. Six of the 7 studies assessed attitudes toward condom use, for which none showed a significant positive effect of serious games [33,40,42-45]. Overall, 3 studies investigated attitudes toward sexual health and sexual behavior in a summary variable [35,37,47], but only Chib’s study [35] observed a positive impact of serious games on participants’ attitudes. Six studies showed positive effects only on specific aspects and 1 study found improved attitudes only for 2 subgroups. Two studies did not demonstrate positive effects on attitudes, but trends indicating a positive impact of serious games emerged. One study provided evidence of positive attitudinal change based on qualitative surveys.

#### Self-efficacy and Personal Limitations

Out of 18 studies, 10 assessed self-efficacy as a dimension of effectiveness. One study reported the self-efficacy dimension in a summary variable, consisting of pubertal support, condom use, contraceptive discussions with partner, and rejection of risky situations [47]. This study found positive effects of serious games on self-efficacy and positive evidence of change in the qualitative surveys and the gaming experience survey [47]. Seven studies analyzed the effects on self-efficacy of condom use and other contraceptive methods in a stand-alone variable [33,40,42-46]. In 6 studies, improvements in self-efficacy related to condom use and contraception were found within the intervention group [33,40,42-44,46]. Another aspect of self-efficacy considered by 7 of the 10 studies was the self-confidence to refuse sexual intercourse in a pressure situation when there is no consent for it [33,40,42-46]. Four of the 7 studies demonstrated positive effects of serious games on self-efficacy to refuse unwanted sex [40,43,45,46]. All 10 studies found a positive effect of serious games on the dimension of self-efficacy, although 5 studies did not show a positive influence of serious games in every aspect of self-efficacy. One study was not able to confirm the positive effects in all cultural subgroups. Related to personal boundaries, 4 studies observed positive effects of serious games and 1 of these studies added positive evidence from a feasibility study.

#### Character Traits and Future Orientation

Two out of the 18 studies examined the effects of serious games on participants’ character traits, such as character qualities (eg, responsibility) and future orientation (eg, having plans for one’s future) [40,42]. One study found positive effects [40], whereas the second study did not demonstrate any effect on participants’ character after using the serious game [42]. Four studies investigated the impact of serious games on future orientations [40,42,45,47]. None of the 4 studies found a significant effect after using the investigated serious game. However, the qualitative study conducted by Winskell et al [47] recorded positive expressions of participants and their parents on the topic of future orientation.

#### Environmental and Individual Risk Factors

The impact of serious games on selected environmental and individual risk factors (eg, exposure to risky situations) has been addressed in 9 studies. Six out of these 9 studies looked at the impact of the serious game on confronting risky situations [40,42-46]. Four studies did not observe a positive effect on this aspect [40,42-44]. Seven of the 9 studies made statements about the influence of serious games on how sexual topics are handled in the personal environment [40-45,47]. Communication about sexual issues with parents was addressed in 5 of the 7 studies [40-44]. In this regard, 1 study found significant improvements in parental communication after the use of a serious game [40]. Three studies showed no positive effects on parental communication [42-44]. In the qualitative survey by Oliveira et al [41], evidence of improved parental communication was shown.

#### Risk Perception and Risk Assessment

Two of the 18 studies examined efficacy in aspects of risk perception and risk assessment. Overall, positive effects on this were found but only to a slightly weak level [38,47]. No positive effects were found on alcohol as a risk factor [43].

#### Normative Beliefs and (Social) Norms

Nine out of the 18 studies examined the influence of serious games on participants’ (perceived) norms. In 6 studies, a positive influence of serious games on the perceived views of those around them about sex was found. Furthermore, in these 6 studies, a positive effect of serious games on perceived views in the personal environment about sexual abstinence was observed [33,40,42,43,45,46]. Among the 9 studies, 4 considered the aspect of perceived norms related to condom use, for which no study found a positive effect of serious games [33,43,44,46]. One of the 4 studies assessed norms related to HIV and STI and found a positive effect of serious games [43].

### Aspects of User/Gaming Experience

#### Motivation

Ten studies considered the aspect of motivation related to the use of serious games. Seven studies asked whether the respondents were willing to recommend the serious game to friends or classmates and in all these studies, the majority of participants would recommend the respective serious game to others [30-35,38,47]. Four of these 7 studies additionally integrated the question whether the participants were willing to repeat the serious game [30,33,38,47], where 3 of them found that a majority of participants were willing to do so [30,38,47]. Three out of the 10 studies addressed the aspect of motivation in general [32,39,41]. One study assessed motivation using questions in the domains of attention, relevance, confidence, and satisfaction related to the serious game, showing increased motivation among intervention participants [39]. Oliveira et al [41] inferred a positive motivational performance in the serious game. Therefore, 10 of 11 studies found a positive motivational performance in the serious game.

#### Acceptability

Acceptability toward the use of serious games has been investigated in 6 studies. In 2 studies, the duration and pace of the intervention was perceived to be appropriate [31,33], which was shown as determinant of acceptability. In the remaining studies, the method for assessing acceptability was not transparent [36,38]. However, these 2 studies indicated that acceptability toward serious games can at least be assumed based on the answers related to the user experience [36,38].

#### Trustworthiness

The trustworthiness of the content of serious games is another aspect that 5 of the 11 studies included in the survey of participants after the intervention was used. Four of the studies refer to the assessment of truthfulness and accuracy [30-33], and 1 study considered only the truthfulness of the information [35]. These 5 studies found that the majority of participants rated the content as credible and accurate [30-33,35].

#### Comprehensibility

Six out of the 11 studies considered the aspect of comprehensibility [30-33,35,39]. Overall, the comprehensibility of the serious games has been judged positively. This relates to the content as well as wording and terminology used in the serious game [30,31,33]. Particularly, qualitative studies described that the participants described the content of the game as easily understandable [39,41].

#### Handling and Control

Five of the 11 studies evaluated the handling and control of serious games. Four studies investigated the aspect of handling, among others, by assessing the difficulty of operation [31,33,35]. This was rated as easy by the majority of participants in all 4 studies. An adult’s assistance in using the serious game was reported in 3 of the 5 studies [30,31,33]. In these 3 studies, it was found that the majority of participants did not require adult assistance.

#### Perceived Effectiveness

Perceived effectiveness is another aspect explored in 10 of the 11 studies. Six studies considered the impact of serious games on perceived decision-making and conflict resolution skills [30-33,36,47]. Here, the majority of participants found the information conveyed through serious games to be helpful in making future decisions regarding sexual issues. Gariepy et al [38] found that most participants felt responsible for the decisions made in the game and the majority would transfer those decisions to real life.

#### Satisfaction

In all 11 studies, statements of satisfaction were recorded. Seven studies asked about personal enjoyment of the game, and the majority of participants indicated to have enjoyed the game and its features [30-34,36,47]. Gariepy et al [38] demonstrated an association between enjoyment and effectiveness outcomes. When comparing traditional school instruction to the use of serious games, participants responded to serious games significantly more positively in almost all cases. The exception was the comparison with their own favorite video game [31-33]. In summary, all 11 studies showed increased satisfaction with the use of the serious game.

## Discussion

### Overview

After reviewing previous studies conducted on serious games in the context of STI prevention, it can be summarized that both effectiveness and user experience have been investigated. However, a stronger focus on the parameters of effectiveness is needed by going beyond feasibility and usability studies and by applying adequate study designs such as RCTs and longitudinal studies. Knowledge about sexual health was the most commonly used dimension in studies investigating the effects of serious games in this area. Almost all studies found a positive effect of the respective serious game on participants’ knowledge. The second most common dimension related to participants’ attitudes and beliefs toward sexuality, sexual behavior, and other sexual topics. Here, just over half of the studies found that participants reported more positive attitudes after using the serious game. The positive effects were not evident in each study for all aspects related to attitudes. Furthermore, long-term effects related to intentions and behaviors related to sexual health need to be investigated.

### Accordance With Previously Published Works

Evidence of nondigital curriculum-based sex and HIV education programs clearly shows that these programs do not hasten or increase sexual behavior. Instead, they either delay sexual behaviors or increase condom or contraceptive use [48]. However, a previous systematic review on digital media to improve adolescent health showed mixed results or effect in unexpected directions [49]. The results of the knowledge, attitudes, and behavior dimensions found in this systematic review on the effects of serious games show similarities to a meta-analysis published in 2015 [24] that investigated the effectiveness of disease prevention and health promotion interventions in the context of sexual health delivered via serious games. This meta-analysis also found an effect of serious games on sexual knowledge. However, no significant effects of serious games were found for the dimension of attitudes and behavior [24]. A parallel can be drawn with the ambiguous attitudinal and behavioral results presented in this systematic review. Further similarities can be seen in the positive effects on self-efficacy. For behavioral intentions, positive effects of serious games have been shown in the meta-analysis by DeSmet et al [24], which are divergent from those reported in this study.

Commonalities between the knowledge, attitudinal, and behavioral dimension results can also be found in a systematic review examining the impact of school-based skill-building behavioral interventions for STI prevention [50]. These behavioral interventions are primarily non–computer-based. Nevertheless, parallels to this work can be seen in the impact dimensions. For example, positive effects on knowledge and self-efficacy can also be found in the review by Picot et al [50]. Positive effects on behavior could only be shown in some studies and for certain aspects and subgroups, as in this work. In both reviews, the effects related to behavior were small. For the results related to attitudes and intentions, both reviews have in common that positive effects have not been observed in all studies [50].

Compared to the results of 3 meta-analyses, the results of this study show similarities and differences. The meta-analyses refer to interventions used to promote sexual health and prevent STIs [51-53]. One meta-analysis for this includes computer-based interventions that do not exclusively target adolescents [51]. The other 2 meta-analyses do not exclusively include computer-based interventions but narrow their analysis to target adolescents [52,53]. Overall, the 3 meta-analyses detected effects of the interventions on knowledge [51-53]. Two meta-analyses also showed effects on self-efficacy and behavioral intentions [51,53], with 1 meta-analysis additionally finding positive effects on attitudes [53]. Positive behavioral effects can be found in all 3 studies [51-53]. Thus, the knowledge and self-efficacy outcomes show parallels to the intervention effects analyzed in this systematic review. Differences in the results appear in the effects on attitudes, intentions, and behavior. For these, no unambiguously positive results can be found in our systematic review compared to the results of the meta-analyses.

Two further meta-analyses identified positive significant effects of computer-based interventions on sexual behavior [54,55]. These publications showed clearer effects of computer-based interventions compared to those shown in this study. However, none of these meta-analyses were limited to adolescents [54,55]. One meta-analysis focused exclusively on HIV prevention interventions [54] and 1 meta-analysis investigated further interventions that aim to promote health-seeking behaviors in addition to interventions related to sexual behaviors [55].

In summary, there is a need for further studies investigating the effects of serious games on knowledge, attitude, and behavior, which go beyond computer-based interventions and which compare their effects with established (nondigital or hybrid) STI prevention strategies among adolescents. Future studies need to consider specific challenges of evaluating impacts of interventions on sexual behaviors among adolescents, particularly among adolescents who may not yet be sexually active. For that reason, longitudinal study designs are needed, which understand serious games as complex interventions within complex systems.

### Limitations in This Review

This systematic review has been able to provide an overview of the current state of evidence of serious games in the context of STI prevention. However, owing to the heterogeneity of studies (eg, differences in interventions, data collection methods, follow-up periods), a meta-analysis was not possible. Moreover, these variations may explain the partly divergent results regarding the effects of serious games. In addition, high attrition, low response rates, or refusal to participate among control and intervention participants, as well as the collection of self-reported information in some studies represent key limitations that must be considered when interpreting the results. Beyond the methodological and content-wise differences that may have influenced the comparison of the studies, there are further limitations related to the conduction of this systematic review. The search was based on 2 databases (PubMed and Web of Science). The dimensions of effects investigated in this systematic review are derived from the parameters used in the primary studies. It should be noted that the authors of the primary studies may have either a different understanding of or used different ways for operationalizing each dimension. An additional limitation is the fact that there are several interventions focusing on the same intervention (“It’s Your Game”), which may also impact the variety of dimensions under consideration in previous studies. Furthermore, it should be noted that all studies found effects of the respective serious game under consideration. It should be questioned to what extent intervention studies that did not find effects for one dimension were not published (publication bias) and, thus, a distorted picture exists regarding the effectiveness of serious games.

### Conclusions

Overall, the effects of serious games in CSE and STI prevention have been shown in this systematic review. However, not all dimensions show comparable effects and some dimensions have only been considered in single or few studies. In addition, the data collection for investigating the effect dimensions is divergent, making comparisons difficult. Nevertheless, it can be stated that above all, knowledge is already comprehensively assessed and a positive influence of serious games on knowledge about sexual topics is evident. In contrast, only limited evidence is available for effects of serious games related to attitudes and behavior. Particularly for sexual behavior, there is a lack of results, as this dimension could only be surveyed in a few studies. This is due to methodological deficits in the surveys, which make it difficult to determine changes in attitudes and behavior, and because sexual topics continue to be taboo in many societies, which limits the ability to survey sexual behavior. According to the results of this systematic review, serious games show potential in the context of STI prevention. Owing to a lack of evidence regarding the effects on attitudes and behavior, no explicit benefit of serious games compared to established (nondigital) methods of STI prevention can be demonstrated. One aspect that should be pursued in this regard is the comparison with classical prevention activities that do not use (digital) media. Until now, there is missing evidence on long-term effects, particularly related to the impacts of serious games on attitudes and behaviors.

## Appendix

Multimedia Appendix 1 Characteristics of the interventions.

## Abbreviations

CSE: comprehensive sexuality education
PRISMA: Preferred Reporting Items for Systematic Reviews and Meta-Analyses
RCT: randomized controlled trial
STI: sexually transmitted infection
