# Supplementary material for: An Evidence Map on Serious Games in Preventing Sexually Transmitted Infections Among Adolescents: Systematic Review About Outcome Categories Investigated in Primary Studies
Source: JMIR Serious Games. 2022 Feb 2;10(1):e30526. doi: 10.2196/30526 (PMC8851332; doi:10.2196/30526)
Supplement: Multimedia Appendix 1 [file games_v10i1e30526_app1.docx]

**Multimedia Appendix 1.** Characteristics of the interventions within primary studies.

| ***Reference*** | ***Intervention*** |
| --- | --- |
| Bertozzi et al [34] | My future family: Video game that educates adolescents about human sexuality and reproduction while asking players when they would like to achieve 5 important family planning milestones. |
| Chib [35] | Planeta Riesgo X: In the educational game via a multimedia CD, 2 cybernetic youth search for new adventures, in the course of which they encounter situations dealing with sexual and reproductive health. |
| Chu et al [36] | Making Smart Choices: Five mini-games offering different scenarios for participants to make decisions using chosen virtual characters. |
| Escobar-Chaves et al [30] | It’s Your Game: Keep It Real: It uses both classroom and computer instruction to promote abstinence and to teach students about their bodies, healthy relationships, personal boundaries, and protecting themselves from pregnancy and sexually transmitted diseases. The curriculum includes 24 lessons (54 minutes each). |
| Fiellin et al [37] | Play Forward: Elm City Stories: Two-dimensional, role-playing adventure video game. The game consists of approximately 16 hours of gameplay in an interactive virtual world. The player’s goal is to acquire and practice skills to reduce risk behaviors and gain knowledge and healthier attitudes and intentions with the ultimate goal of HIV prevention. |
| Gariepy et al [38] | Mobile videogame intervention to decrease high-risk sexual behavior in Black and Hispanic adolescents. |
| Haruna et al [39] | Game-Based Learning in which digital stories were woven into the learning content and embedded in an interactive manner. |
| Markham et al [40] | It’s Your Game: Keep It Real: It uses both classroom and computer instruction to promote abstinence and to teach students about their bodies, healthy relationships, personal boundaries, and protecting themselves from pregnancy and sexually transmitted diseases. The curriculum includes 24 lessons (54 minutes each). |
| Oliveira et al [41] | Papo Reto: Online game using a scenario of a city in which various spaces can be progressively accessed (home, school, internet, clubs and street) and the players respond to situations and interact with his/her peers. |
| Peskin et al [42] | It’s Your Game: Keep It Real: It uses both classroom and computer instruction to promote abstinence and to teach students about their bodies, healthy relationships, personal boundaries, and protecting themselves from pregnancy and sexually transmitted diseases. The curriculum includes 24 lessons (54 minutes each). |
| Peskin et al [43] | It’s Your Game: Keep It Real: It uses both classroom and computer instruction to promote abstinence and to teach students about their bodies, healthy relationships, personal boundaries, and protecting themselves from pregnancy and sexually transmitted diseases. The curriculum includes 24 lessons (54 minutes each). |
| Potter et al [44] | It’s Your Game-Tech: Completely computer-based, middle school sexual health education program. The It’s Your Game-Tech curriculum comprises 13 lessons (35-45 minutes each). The curriculum is set within a mall-like environment that includes several “storefronts” and “proprietors.” Within the environment, students are guided by 2 animated narrators who introduce selected activities (eg, animated scenarios with modeling and skills practice, peer modeling videos, quizzes, fact sheets, a graffiti wall for personalization and reflection, “point of view” virtual role-play activities that simulate student skills practice in real-world situations). |
| Rohrbach et al [45] | It’s Your Game: Keep It Real: It uses both classroom and computer instruction to promote abstinence and to teach students about their bodies, healthy relationships, personal boundaries, and protecting themselves from pregnancy and sexually transmitted diseases. The curriculum includes 24 lessons (54 minutes each). |
| Shegog et al [33] | It’s Your Game-Tech: Completely computer-based, middle school sexual health education program. The It’s Your Game-Tech curriculum comprises 13 lessons (35-45 minutes each). The curriculum is set within a mall-like environment that includes several “storefronts” and “proprietors.” Within the environment, students are guided by 2 animated narrators who introduce selected activities (eg, animated scenarios with modeling and skills practice, peer modeling videos, quizzes, fact sheets, a graffiti wall for personalization and reflection, “point of view” virtual role-play activities that simulate student skills practice in real-world situations). |
| Shegog et al [31] | Native It’s Your Game: Adaptation of the technology-based sexual health curriculum “You’re your Game.” Completely computer-based, middle school sexual health education program. The It’s Your Game-Tech curriculum comprises 13 lessons (35-45 minutes each). The curriculum is set within a mall-like environment that includes several “storefronts” and “proprietors.” Within the environment, students are guided by 2 animated narrators who introduce selected activities (eg, animated scenarios with modeling and skills practice, peer modeling videos, quizzes, fact sheets, a graffiti wall for personalization and reflection, “point of view” virtual role-play activities that simulate student skills practice in real-world situations). |
| Shegog et al [32] | Secret of Seven Stones: Web-based sexual health intergenerational adventure game |
| Tortolero et al [46] | It’s Your Game: Keep It Real: It uses both classroom and computer instruction to promote abstinence and to teach students about their bodies, healthy relationships, personal boundaries, and protecting themselves from pregnancy and sexually transmitted diseases. The curriculum includes 24 lessons (54 minutes each). |
| Winskell et al [47] | Tumaini: Tumaini ("hope for the future" in Swahili) is an interactive, narrative-based game. It uses interactive narratives to promote observational learning, cognitive and behavioral rehearsal, problem-solving, and immersion. |
